# Health Behaviors: Is There Any Distinction for Teachers? A Cross-Sectional Nationwide Study

**DOI:** 10.1371/journal.pone.0120040

**Published:** 2015-03-20

**Authors:** Fabien Gilbert, Jean-Baptiste Richard, Pascale Lapie-Legouis, François Beck, Marie-Noël Vercambre

**Affiliations:** 1 MGEN Foundation for Public Health, Paris, France; 2 National Institute for Prevention and Health Education (INPES), St Denis, France; 3 French Monitoring Center for Drugs and Drug Addiction (OFDT), St Denis, France; 4 Centre de recherche Psychotropes, Santé mentale, Société (CESAMES), CNRS UMR 8136, INSERM U611, Université René Descartes Paris V / Sorbonne Paris Cité, Paris, France; Örebro University, SWEDEN

## Abstract

**Background:**

Health behaviors, as important modifiable determinants of health, are consistently targeted by prevention messages. Teachers, as educators and role models, may play a key-role in bringing such messages to children and adolescents. It is not clear which areas of prevention could be improved in collaboration with teachers to promote healthy behaviors at the population level through health education in schools.

**Methods:**

to evaluate teacher’s health awareness, we compared their health/risk behaviors to those of non-teachers, taking into account demographic and socioeconomic factors that could confound crude differences. We used data from the 2010 Health Barometer, a cross-sectional nationally-representative French survey conducted by telephone among 27,653 persons aged 15–85. Adjusting sequentially for potential confounders, we compared six indicators of lifestyle and risky conducts (at-risk drinking, current smoking, cannabis use, gambling, corpulence, sleep duration) between teachers (n = 725) and two comparison groups: other occupations (n = 12,483) on the one hand, and other intermediate and managerial/professional occupations (n = 6,026) on the other.

**Results:**

In the fully-adjusted models, teachers were less likely than other occupations to smoke, to have used cannabis in the last 12 months, to gamble regularly and to be overweight or obese. When restricting the comparison group to other occupations belonging to the same socio-professional category, differences were attenuated, but remain highly significant for tobacco, cannabis and gambling. No significant differences were observed between teachers and non-teachers regarding alcohol use and sleep duration, once important confounders had been adjusted for.

**Conclusions:**

Our results suggest that teachers behave on the whole more healthily than other adults with a similar demographic and socioeconomic profile. The absence of a teacher distinction toward at-risk drinking needs to be examined in more detail.

## Introduction

Health behaviors are important risk factors for major chronic diseases such as cardiovascular diseases or cancers [1, 2] and for this reason are regular targets of public health messages. An important channel of communication for such messages is the schoolroom [3–5]. Indeed, teachers play a key role in health education for the young, both through providing information and knowledge and through serving as role models for their pupils [6, 7]. However, teachers may feel ill at ease with certain health topics or certain health education approaches [8]. They also may be indifferent to such interventions [9]. To identify health education area in which to invest in collaboration with teachers to promote healthier lifestyles at the population level [10, 11], it is of interest to better characterize their health awareness, both as a basis on which to build effective health education strategies and as an indicator of propensity to be involved in them. To do so, one possible approach is to study their health behaviors.

The literature available on teacher’s health behaviors is scarce and inconsistent. For example, some studies on tobacco use have found a lower smoking prevalence in teachers compared to the general population [6, 12–14], whereas other studies did not [15, 16]. Overall, indicators of health behaviors that have been considered were inconsistently defined across studies and comparison groups have been heterogeneous. Moreover, most studies did not adjust for demographic, socioeconomic and health factors, although differences in these factors may possibly account for entirely any differences observed in health behaviors [17–19]. Regarding health outcomes, some evidence suggest that teachers in Europe represent a particularly healthy population: they show comparatively low death rates [20], report a high health-related quality of life [21, 22] and do not have higher rates of mental disorders [23]. Although this could be related to healthier behaviors among teachers, it is impossible to conclude without further investigation. Indeed, health is highly multifactorial, and other factors such as more favored social origins as well as more auspicious socioeconomic/occupational conditions could greatly contribute to the good health status of teachers.

Our hypothesis was that teachers behave on the whole more healthily than other workers, and that this difference could not be explained completely by their relatively higher socioeconomic status. This assumption is sustained by the facts that teachers are not only learned, but also, as educators, have (or believe they have) moral obligation to promote healthy behaviors through exemplarity. To test our “healthy teacher distinction” hypothesis, we used data from a nationally-representative cross-sectional French survey conducted by telephone in 2010 which involved 27 653 adults. We compared various indicators of lifestyle and risk behaviors between teachers and two comparison groups, namely all other occupations and other intermediate and managerial/professional occupations. We adjusted progressively for demographic and socioeconomic factors that could confound interpretation of any potential differences in health behaviors observed.

## Methods

### The 2010 Health Barometer

The 2010 Health Barometer is a nationally-representative telephone survey on health behaviors and attitudes toward health carried out from October 2009 to July 2010 by the French National Institute for Prevention and Health Education (INPES) [24] [25]. The survey conformed to the principles embodied in the Declaration of Helsinki and was approved by the French National Commission for Data Protection and Liberties (CNIL). It was performed using a Computer-Assisted Telephone Interviewing (CATI) system and was based on a two-stage (household then individual) random sampling method. Telephone numbers were generated randomly so that households corresponding to unlisted phone numbers were included. Before the first telephone call (or after the first call if the address could not be retrieved using the reverse directory), households selected at random were sent a mail with a full description of the study objectives and a formal request to participate. To be eligible, a household should be reachable by telephone (through a landline or a mobile line) and include at least one French-speaking person aged 15–85. Among these eligible persons in the household, the person surveyed was randomly selected using the objective selection method proposed by Kish [26]. The refusal rate was 40% for both mobile and landline samples. The average questionnaire completion time was 32 minutes. After completion of the interviews, telephone numbers were deleted to protect anonymity and respect privacy. In particular, information was anonymized and de-identified prior to analysis.

### Study sample

Of the 27,653 persons aged 15–85 interviewed in the 2010 Health Barometer, 14,811 were currently in employment. Of these, those aged < 25 or ≥ 65 were excluded (n = 911), as well as those whose teaching status was uncertain (n = 26) and those with at least one missing values for any of the covariates (n = 148) or outcomes of interest (n = 518) assessed. The final analysis sample was comprised of 13,208 employed persons aged 25–64. Of these, 6,751 had intermediate or managerial/professional occupations, of whom 725 were teachers.

### Weighting procedure

The 2010 Health Barometer data were first weighted by the ratio of the number of eligible individuals to the number of telephone lines in a household. Since our study concerned only people in employment, data for employed participants were then weighted in order to match the 2010 French working population structure with respect to gender, age and socio-professional category. To optimize representativeness within the teacher sample, the socio-professional category variable was based on the standard classification in France crossed with the teaching status. As teachers are categorized among the “managerial and professional occupations” (most secondary and higher education teachers) or among the “intermediate occupations” (most primary teachers), we considered the following nine-category classification in the weighting procedure: 1) farmers; 2) self-employed craftsmen, tradesmen and businessmen; 3) non-teaching managerial and professional occupations; 4) non-teaching intermediate occupations; 5) primary teachers; 6) secondary teachers; 7) higher education teachers; 8) employees; 9) manual workers. The 2010 reference data for the whole working population were drawn from the National Institute of Statistics and Economic Studies [27] and those specific to the teacher subpopulation from the French Ministry of Education [28].

### Health behavior indicators

As outcomes, we studied six indicators of health behavior: three related to psychoactive substance use (alcohol, tobacco and cannabis) and three related to lifestyle (gambling, body mass index and sleep habits). Our choice of these outcomes was guided by two criteria: firstly the indicator should be a significant risk factor for physical or mental health and secondly reliable information about it should be available in the 2010 Health Barometer. Of these outcomes, four were defined as a binary variable: risk drinker (according to the Alcohol Use Disorder Identification Test—AUDIT [29]) versus no-risk or non-drinker; current smoker versus non-smoker; past-year user of cannabis versus non-user; regular gambler (plays games by chance for money more than weekly on average or for more than 500€ per year) versus no or occasional gambler. The two further outcomes were defined as three-category variables, with the intermediate category being the reference, also presumably the healthier category. Thus, body mass index (BMI) was used as an indicator of corpulence related to the balance between food intake and energy expenditure [30] and classified as underweight (BMI ≤18 kg/m^2^), normal range of weight (BMI 18–25 kg/m^2^) or overweight or obese (body mass index ≥ 25). Similarly, sleep duration was used as an indicator of healthy sleep habits [31] and was classified as short sleeper (usual sleep duration per night ≤ 6 hours), normal sleeper (usual sleep duration 6–9 hours), long sleeper (usual sleep duration > 9 hours).

### Covariates

To limit confounding bias, we considered information on demographic, socioeconomic and health-related factors plausibly linked with both health behaviors and teaching status (teacher: yes/no). We followed a hierarchical approach to assess the relative importance of different types of confounding factors. The basic model (M1) included only demographic factors (gender and age) as adjustment variables. The second model (M2) also considered socioeconomic factors (education, self-perceived financial situation, marital status, presence at home of a child aged <18 years, and type of community). Finally, in the third model (M3), we also introduced two general health indicators as adjustment variables. These were self-reported chronic disease (yes/no) and the Duke Health Profile, a 17-item instrument that measures perceived general health, as an indicator of bad health [32, 33]. This latter score was dichotomized at 60, which corresponded to the first quintile of the whole population.

### Statistical analysis

In a first step, we evaluated the representativeness of our sample of participant teachers compared to the national teaching force with respect to key sociodemographic characteristics using data from the French Ministry of Education [28]. Next, and using chi2 test, we compared the characteristics of teachers (n = 725) to two other non-teaching employment groups in order to document the demographic, socio-economic and general health characteristics of the teaching profession. These were firstly all other adults in employment (n = 12,483) and secondly other intermediate and managerial/professional occupations (n = 6,026). The latter group was chosen to refine the analysis by limiting socioeconomic heterogeneity.

For each of the six indicators of health behavior, we used binomial or multinomial logistic regression models to contrast teachers with the two comparison groups adjusting sequentially for three blocks of major confounding factors, namely demographic factors (M1), socioeconomic factors (M2) and health-related factors (M3). We evaluated the strength of association using odds ratios together with their 95 percent confidence intervals (OR [95%CI]). A probability threshold of 0.05 was considered statistically significant and all statistical tests were two sided. All analyses were performed using Stata SE 12 software.

## Results

### Demographic, socioeconomic and health specificities of teachers

After weighting, our sample of teachers was similar to that of the national education system reference with respect to gender, age and teaching level (Table 1). In particular, the data illustrate that the teaching profession is predominantly female and highly educated (Table 2). Overall, 67% of teachers in our sample were women compared to 48% among non-teaching occupations and 44% among non-teaching intermediate/professional occupations. Moreover, 88% of teachers had at least an undergraduate degree compared to 19% for all other occupations and 40% for other intermediate/professional occupations. Also, teachers were less likely than other workers to think that their financial situation was a difficult one (4% vs. 12% and 6%). Regarding health indicators, the prevalence of self-reported chronic disease was similar between teaching and non-teaching occupations (17% vs. 16% and 16%), and the prevalence of a low perceived health score was intermediate (13% vs. 15% and 10%).

**Table 1:** Key characteristics of teachers interviewed in the 2010 Health Barometer: raw and weighted^a^ data and comparison to national statistics for the whole teaching force.

		Teachers interviewed in the Health Barometer 2010		All teachers in the national education system
		Raw data	Weighted data	Reference^b^
Teaching level (%)	Primary education	44%	40%	40%
	Secondary education	49%	52%	52%
	Higher education	7%	8%	8%
Proportion of women (%)	Primary education	84%	82%	83%
	Secondary education	64%	59%	59%
	Higher education	48%	39%	37%
	Total	71%	67%	67%
Mean age (SD) in years	Total	42 (10)	42 (10)	43 (-)

^a^ Weighting procedure taking into account the ratio of the number of eligible individuals to the number of telephone lines in a household, as well as gender, age and socio-professional category

^b^ National statistics from the reference book annually published by the French Ministry of Education (RERS 2010); SD for age not available

**Table 2:** Characteristics of teachers compared to two groups of other occupations, 2010 Health Barometer.

	Teachers	Other occupations	
		All	Intermediate, managerial/professional
	% (N = 725)	% (N = 12,483)	% (N = 6,026)
*Gender*		***	***
Male	33.2	52.4	55.8
Female	66.8	47.6	44.2
*Age*		*ns*	*ns*
25–34 years	28.0	25.3	27.1
35–44 years	34.0	31.9	33.5
45–54 years	24.6	29.6	26.4
55–64 years	13.4	13.2	13.0
*Education*		***	***
High School diploma	12.4	80.7	60.3
Undergraduate degree	62.9	9.0	17.1
Postgraduate degree	24.7	10.3	22.6
*Self-perceived financial situation*		***	*
Comfortable / ok	76.6	60.9	72.9
A bit short of money	19.8	27.2	21.0
Tough / in debt	3.6	11.9	6.1
*Socio-professional category*		***	*ns*
Farmers	0.0	2.2	0.0
Craftsmen and traders	0.0	7.4	0.0
Managerial/professional	39.2	17.0	41.4
Intermediate	60.8	24.1	58.6
Employees	0.0	29.5	0.0
Manual workers	0.0	19.8	0.0
*Marital status*		*	*
Married or civil union	66.1	61.4	63.4
Single / widow(er)	25.9	31.6	30.6
Divorced	8.0	7.0	6.0
*Child < 18 years living at home*		*	*
Yes	57.4	52.5	53.1
No	42.6	47.5	46.9
*Type of community*		*	***
Rural	29.2	31.9	24.8
2000–199,999 inhabitants	34.6	34.7	32.6
≥ 200,000 inhabitants	24.6	19.5	23.1
Paris area	11.6	13.9	19.5
*Self-reported chronic disease*		*ns*	*ns*
Yes	17.0	15.7	15.6
No	83.0	84.3	84.4
*Duke Health Profile score*		*ns*	*ns*
Low (< 60)	12.5	14.8	10.2
Normal to high (≥ 60)	87.5	85.2	89.8

*,*** distribution significantly different from teacher’s at 5% level and 0.1% level respectively (p-value from chi 2 test)

### Health behaviors of teachers compared to non-teachers

When successively modelling the six indicators of health behavior studied, and after adjusting on various demographic, socioeconomic and health related factors (M3), we found that teachers, compared to other occupations, were less likely to smoke (OR [95% CI] = 0.59 [0.47–0.74], p<0.01) and to have used cannabis in the past year (OR [95% CI] = 0.49 [0.30–0.82], p<0.01) (Table 3). They were also less prone to gamble regularly (OR [95% CI] = 0.41 [0.25–0.68], p<0.01) and less likely to be overweight (OR [95% CI] = 0.77 [0.62–0.96], p = 0.02).

**Table 3:** Health behavior of teachers compared to those of two groups of other occupations, basic and further adjustment, 2010 Health Barometer.</

Health behavior: outcome	Teachers	All other occupations (G1)	Other intermediate and managerial professional occupations (G2)		Teachers vs. G1		Teachers vs. G2	
	N = 725	N = 12,486	N = 6,026	Model type^a^	OR (95% IC)^b^	p-value	OR (95% IC) ^b^	p-value
Tobacco^c^: current smoker	21.5%	36.3%**	32.3%**	M1	0.49 [0.40–0.60]	<0.01	0.59 [0.48–0.73]	<0.01
*versus non-smoker*				M2	0.60 [0.48–0.75]	<0.01	0.63 [0.50–0.79]	<0.01
				M3	0.59 [0.47–0.74]	<0.01	0.62 [0.49–0.78]	<0.01
Alcohol ^c^: risk drinker	10.1%	14.3%**	14.3%**	M1	0.87 [0.65–1.16]	0.33	0.87 [0.65–1.17]	0.36
*versus no-risk or non-drinker*				M2	0.86 [0.63–1.18]	0.36	0.88 [0.64–1.20]	0.40
				M3	0.86 [0.63–1.17]	0.34	0.86 [0.63–1.18]	0.35
Cannabis^c^: past year-user	3.0%	5.5%**	6.3%**	M1	0.63 [0.39–0.99]	0.05	0.58 [0.36–0.93]	0.02
*versus non-user*				M2	0.49 [0.30–0.82]	<0.01	0.52 [0.31–0.87]	0.01
				M3	0.49 [0.30–0.82]	<0.01	0.52 [0.31–0.87]	0.01
Gambler ^c^: regular	2.9%	12.5%**	9.8%**	M1	0.23 [0.14–0.37]	<0.01	0.32 [0.20–0.51]	<0.01
*versus occasional or non-gambler*				M2	0.41 [0.25–0.68]	<0.01	0.46 [0.27–0.77]	<0.01
				M3	0.41 [0.25–0.68]	<0.01	0.46 [0.27–0.76]	<0.01
Corpulence ^d^: BMI ≤18	5.4%	2.8%*	3.2%	M1	1.27 [0.87–1.86]	0.22	1.15 [0.78–1.71]	0.48
*versus 18*.*1–24*.*9*				M2	1.40 [0.91–2.16]	0.13	1.40 [0.90–2.17]	0.14
				M3	1.40 [0.91–2.15]	0.13	1.38 [0.89–2.14]	0.15
Corpulence ^d^: BMI ≥ 25	24.0%	39.5%**	35.3%**	M1	0.57 [0.47–0.70]	<0.01	0.74 [0.60–0.92]	<0.01
*versus 18*.*1–24*.*9*				M2	0.78 [0.63–0.97]	0.02	0.85 [0.68–1.07]	0.16
				M3	0.77 [0.62–0.96]	0.02	0.84 [0.67–1.05]	0.13
Sleep duration ^d^ ≤ 6 h per night	13.6%	21.4%**	17.8%**	M1	0.62 [0.49–0.78]	<0.01	0.81 [0.63–1.03]	0.08
*versus >6–9 h*				M2	0.86 [0.67–1.12]	0.28	0.94 [0.72–1.23]	0.63
				M3	0.86 [0.66–1.11]	0.24	0.92 [0.70–1.21]	0.56
Sleep duration ^d^ > 9 h per night	2.0%	3.6%*	2.4%	M1	0.45 [0.23–0.87]	0.02	0.66 [0.34–1.30]	0.23
*versus >6–9 h*				M2	0.75 [0.37–1.52]	0.43	0.88 [0.43–1.81]	0.73
				M3	0.75 [0.37–1.51]	0.43	0.87 [0.42–1.77]	0.69

BMI: body mass index (in kg/m^2^); World Health Organization categories: underweight if BMI ≤18, overweight if BMI ≥ 25, obese if BMI ≥ 30.

^a^ M1: adjusted for age and gender; M2: further adjusted for education, self-perceived financial situation, presence at home of a child aged less than 18 years and type of community; M3: further adjusted on self-reported chronic disease and indicator of bad health based on the Duke Health Profile score; All the adjustment variables were categorized as documented in Table 2.

^b^ From logistic regressions

^c^ Dichotomous outcomes were modeled using binomial logistic regressions

^d^ Polytomous outcomes were modeled using multinomial logistic regressions

*,** Prevalence significantly different from teacher’s at 5% level and 1% level respectively

The prevalence of risk drinking and both short and long sleep duration were lower for teachers than for non-teachers. However, these differences were no longer significant when taking into account important confounding factors, in particular gender and age for alcohol consumption (association no longer significant in M1) and socioeconomic factors for sleep duration (associations still significant in M1 but no longer significant in M2).

When comparing teachers to other intermediate and managerial/professional occupations (Table 3), the differences in behaviors toward tobacco, cannabis and gambling were reduced but still remained highly significant (p≤0.01). Compared to other intermediate and managerial/professional occupations, teachers did not differ with respect to alcohol consumption, BMI and sleep duration.

## Discussion

In our multi-adjusted comparison study of six indicators of health behavior, teachers were less likely than other workers to smoke, to have used cannabis in the past year, to gamble regularly and to be overweight or obese. When restricting the comparison group to other occupations belonging to the same socio-professional category (the intermediate and managerial/professional occupations), these differences were attenuated. The difference in BMI was no longer significant but those regarding tobacco, cannabis and gambling remain highly significant. No differences were observed between teachers and non-teachers regarding alcohol consumption and sleep duration once important confounders such as demographic and socioeconomic factors were taken into account.

On the whole, our findings are consistent with the “healthy teacher distinction” hypothesis: as compared to employed persons with similar socioeconomic profile, teachers were more likely to have healthier behaviors, at least in certain domains, such as tobacco, cannabis, and gambling. These associations between healthier behaviors and teaching status may be related to a higher level of health awareness among teachers, which could be underpinned by two mutually enhancing mechanisms [18]. Firstly, it is possible that those choosing a teaching career may be more health-conscious than those who do not, perhaps through self-selection by shared personality traits, beliefs and values, as well as by social origins, that would influence health behaviors [34]. Secondly, once individuals have entered the teaching profession, they may be prompted (consciously or unconsciously) to conform to healthy norms and internalize positive attitudes towards health and negative attitudes towards health risks [35]. Overall, it would be interesting to assess to what extent teachers feel that they are entrusted with a responsibility for health education of the young. Such a motivation could partly explain their tendency to endorse healthy behaviors as role models.

In contrast to the situation with tobacco and cannabis, the absence of a teacher distinction regarding alcohol consumption is of note and we estimated that one in ten teachers reported at-risk alcohol consumption. The link between alcohol and the school environment, notably with respect to specific work-related stressors and culture, should be examined in more detail [36]. A hypothesis, that remains to be tested, would be that alcohol use, which generally takes place in the private sphere outside school hours and is thus not visible at school, would be less stigmatized than tobacco and cannabis, at least in the educational community. Both the substantial prevalence of at-risk drinking in teachers and the absence of a teacher distinction for alcohol suggest that providing better information on the risks associated with excessive drinking is an important target for a holistic prevention approach in the educational community.

After adjusting for potential confounders, we did not observe significant differences in sleep duration between teachers and other workers. Although sleeping enough is important to be fit, sleep needs greatly differ across individuals [37]. Moreover, sleep duration has been shown to vary by occupation, with work affecting sleep through various factors, including long/extended work hours and other job-related stressors [38, 39]. Therefore, normal sleep duration may not be the best indicator for evaluating sleep hygiene. Another point is that sleep habits, like alcohol consumption, are less visible at school than smoking habits or BMI. Thus, setting an example with respect to sleep hygiene would be less relevant for teachers.

Teachers were less likely to be obese than other workers, but the difference was not significant when restricting the comparison group to other intermediate and managerial/professional occupations. This observation points at the image issue and stigmatization risk in the work environment. In fact, teachers are particularly exposed in the classroom, and appearance, including corpulence, may be considered as crucial to limit stigmatization and achieve teaching effectiveness. However, the absence of a teacher distinction toward BMI among the intermediate and managerial/professional occupations suggests that interaction with the public and exemplarity duty are not the sole factors that contribute to relatively low obesity rates among employees. It would be interesting to examine in-depth the associations between BMI, jobstrain, job insecurity and sector type.

Classically, health behaviors are studied to identify populations at risk or more prone to be involved in risky behaviors. Here, the approach is quite different. We aimed to evaluate to what extent teachers’ health behaviors differ from those of other occupations, considering such health behaviors as valuable indicators of health awareness. Little information was previously available about this topic, although it could clearly contribute, as part of a global health education approach, to establishing more relevant and effective teacher preparation programmes [40]. Teachers are well-placed to play a role in health education due to their familiarity and proximity with the young which is a prerequisite to delivery of health promotion messages toward children and adolescents [41]. For this to be effective, teachers should first of all be well-informed themselves and also encouraged to put into practice their health knowledge. This could perhaps be implemented through training programmes aimed at enhancing the role of teachers as health educators and empowering them. This would be a necessary first step to change behavior effectively among their pupils and students [10].

The strength of our study rests on the quality and richness of the source data obtained through a nationally-representative telephone survey using validated epidemiologic instruments. In particular, we were able to take into account important demographic and socioeconomic confounders. Such adjustment was crucial to identify the specific association of being a teacher with the health-related variables analyzed [18, 42].

However, we were not able to explore certain critical areas of disease prevention and health promotion (notably, physical exercise, which is known to be an important determinant of health risk [43, 44]) nor were we able to explore heterogeneity of health behaviors between teachers. In fact, the data required for such analyses were not available in the Health Barometer survey. Another limitation of the study was that our indicators of health behaviors were based on self-report, and the reliability of the data so obtained may be affected both by selective response and by distorted response among participants. First, and due to non-response, prevalences of at-risk behaviors may have been underestimated. Indeed, some evidence supports that respondents in population-based survey are healthier than non-respondents [45–47]. Consistently, in the 2010 Health Barometer, teachers were more likely to respond than other workers. Although data were weighted taking teaching status, socio-economic and demographic characteristics into account, this is not sufficient to exclude the risk of a residual “healthy respondent” bias, where those who answer the survey have healthier behaviors than those who decline. However, it would not be a problem in this association study, unless this bias is differential according to teaching status, which we have no reason to believe. Second, we cannot exclude a social desirability bias, whereby teachers would be less prone than other adults to report unhealthy behaviors, which could confound, at least in part, the observed associations [48]. Yet, differential desirability bias, if present, would be of low magnitude, as telephone survey provides more anonymity than face-to-face interviews. Moreover, the eventual tendency of interviewed teachers to conform in their responses to the social ideal of their professional category would be limited as the job question was put at the very end of the survey, along with the other socioeconomic items. Finally, given the cross-sectional design of our study, associations between teaching status and health behavior should not be interpreted in terms of causality.

In conclusion, this study examined teachers’ health behaviors and provides a first step toward understanding teachers’ attitudes towards health. Such knowledge is a prerequisite to improve health education strategies at school through a more effective involvement of teachers. The results suggest a relatively high level of health awareness but also identify alcohol consumption as a potential area for improvement. Such data can be useful in designing health education materials that better match the needs of teachers as health educators. A challenge for future research would be to evaluate teacher-pupil relationships better and to understand how this relationship may promote healthy behaviors in pupils.
